# Implementing child and youth mental health services: early lessons from the Australian Primary Health Network Lead Site Project

**DOI:** 10.1186/s13033-021-00440-8

**Published:** 2021-02-23

**Authors:** Sanne Oostermeijer, Bridget Bassilios, Angela Nicholas, Michelle Williamson, Anna Machlin, Meredith Harris, Philip Burgess, Jane Pirkis

**Affiliations:** 1 The University of Melbourne, Melbourne, VIC 3010 Australia; 2 The University of Queensland, Brisbane, Australia

**Keywords:** Mental health, Health care reform, Service uptake, Service implementation, Adolescence, Primary care, Child and adolescent mental health, Australia

## Abstract

**Aim:**

Primary mental health care services play an important role in prevention and early intervention efforts to reduce the prevalence and impact of mental health problems amongst young people. This paper aimed to (1) investigate whether mental health services commissioned by Australia’s 31 Primary Health Networks provided accessible care and increasingly reached children and youth across Australia, and (2) identify the challenges of, and facilitating factors to, implementing services for youth with, or at risk of, severe mental illness (i.e., youth enhanced services) in 10 PHNs which acted as mental health reform leaders (i.e., Lead Sites).

**Methods:**

We used mixed methods, sourcing data from: a national minimum data set that captured information on consumers and the services they received via all 31 PHNs from 1 July 2016 to 31 December 2017; consultations with Lead Site staff and their regional stakeholders; and observational data from two Lead Site meetings.

**Results:**

Many children and youth receiving services were male and up to 10% were Aboriginal and/or Torres Strait Islander young people. The majority of young people came from areas of greater disadvantage. For most children and youth receiving services their diagnosis was unknown, or they did not have a formal diagnosis. Both child and youth service uptake showed a modest increase over time. Six key themes emerged around the implementation of youth enhanced services: service access and gaps, workforce and expertise, funding and guidance, integrated and flexible service models, service promotion, and data collection, access and sharing.

**Conclusions:**

Early findings suggest that PHN-commissioned services provide accessible care and increasingly reach children and youth. Learnings from stakeholders indicate that innovative and flexible service models in response to local youth mental health needs may be a key to success.

## Background

Child and youth mental health problems are an important global public health concern, affecting 10–20% of young people worldwide [1]. Primary mental health care services play an important role in prevention and early intervention efforts to reduce the prevalence and impact of mental health problems amongst young people. This paper describes children and youth who received mental health services, and characteristics of these services, following an Australian mental health care reform in the primary health care setting. Furthermore, it discusses main challenges of, and facilitating factors to, implementing services for youth with, or at risk of, severe mental illness in 10 regions that were selected to act as mental health reform leaders.

## Child and youth mental health in Australia

Young people typically include those aged 10–24 years (according to the World Health Organisation) or a subgroup within this cohort. However, there are various terms to refer to young people of different ages which are often used interchangeably and inconsistently. Other terms may include children (typically under 18 or younger), adolescents, young adults or youth. In Australia, a common distinction is made between ‘children’ aged 0–11 years old and ‘youth’ aged 12–25 years old. For example, the *headspace* National Youth Mental Health Foundation and the Victorian Health Promotion Foundation focus their mental health advocacy, research and/or service activities specifically on young people aged 12–25 years. Throughout this paper we refer to ‘young people’ as those aged 0–25 years old, ‘children’ as those aged 0–11 years and ‘youth’ as those aged 12–25 years.

In Australia, the 12-month prevalence of mental health problems among young people aged 4–17 years has been estimated at 13.9% [2]. The estimates for older young people aged 16–24 years are higher (26.4%), representing the highest prevalence of all age groups [3]. Anxiety and depressive disorders are the second leading cause of total disease burden among young people aged 5–24 years [4].

Young people have been shown to access mental health services less frequently than the overall population [5, 6]. In general, youth accessing services are mostly female, diagnosed with mood and/or anxiety disorders, and reside in major cities [7–9]. Service uptake among children seems to be lower compared to youth and is almost equal across boys and girls [2]. Children accessing services are also mostly diagnosed with mood and/or anxiety disorders and reside in major cities [2].

It has been argued that young people worldwide show lower service access because they are reluctant to seek help for mental health problems and/or because mental health services do not always meet their needs [10]. The main barriers to using services reported by young people in Australia include perceived stigma and embarrassment, fears regarding confidentiality and lack of trust, poor mental health literacy, and a preference for self-reliance [2, 11, 12]. Less is known about facilitating factors, however positive past experience with help-seeking has been identified as one prominent facilitating factor for youth to access mental health services [11]. Youth health workers, general practitioners (GPs) and community health centre staff have reported several challenges in working with youth [13]. These include the need for longer consultations, different communication styles and involving parents. Other challenges include poor linkages between services and inflexible service provision [13].

## Primary mental health services in Australia

In 2015, in response to a major review [14], the Australian Government established 31 Primary Health Networks (PHNs) nationwide to reshape the delivery of health services. PHNs are independent organisations funded by the Australian Government, each operating in their own local regions across metropolitan and rural areas. They are expected to improve integration between services across the entire health system, respond to local needs and improve consumer outcomes [15]. PHNs are to achieve this by understanding the needs of their communities, supporting GPs and other primary care providers in a variety of ways so that they can offer optimal care, and by purchasing or commissioning services. From July 2016, PHNs received funding from the Australian Government Department of Health to plan and commission services in six national mental health priority areas (‘PHN-led reform’), including child and youth services [16], the focus of this paper.

Prior to the PHN-led reforms, the Australian Government funded Medicare Locals to provide primary mental health care via the Access to Allied Psychological Services (ATAPS) program (July 2001 to June 2016) to improve access to services for common mental health problems. Over time ATAPS increasingly targeted hard-to-reach groups (e.g., people at risk of suicide, in rural and remote areas, with perinatal depression, affected by natural disasters) by offering unique flexibilities in service delivery [7]. The ATAPS program included specific services for children and youth with, or at risk of, mental illness [7, 17]. The PHN-led reforms have built on and superseded ATAPS. As part of the PHN-led reforms, PHNs are expected to commission ATAPS-like mental health services according to the needs of their local communities across the lifespan. Examples of mental health services commissioned by the PHNs include psychological therapy, clinical care coordination services, Aboriginal and Torres Strait Islander mental health services and youth-specific mental health services.

Many of the mental health services for youth are delivered through *headspace* centres that specifically target youth aged 12 to 25 years and all PHNs are expected to maintain standard *headspace* services in their regions [18]. Additionally, PHNs can choose to commission *headspace* centres to deliver specialised services (as described below). It should be noted that not all *headspace* services for young people are commissioned by the PHNs.

## Youth enhanced services commissioned by PHNs

PHNs are required to commission enhanced services to meet the needs of youth with, or at risk of, *severe* mental illness but have flexibility to determine how these services are delivered in their region based on local needs. Newly developed models are expected to be in line with current evidence and best practice. Furthermore, these services are expected to offer psychological therapy and vocational support, and involve a broad workforce, such as allied health providers and case managers. Ten PHNs were selected to act as mental health reform leaders (‘Lead Sites’) in several nominated key focus areas via the ‘PHN Mental Health Reform Lead Site Project’ (the ‘Lead Site Project’), including enhanced services for youth. They are spread out across six different states and territories. Seven Lead Sites were located in a metropolitan area and three Sites were based in a rural area. Three of these Lead Sites were specifically selected to focus on enhanced activities in this key focus area.

The Department of Health commissioned our research team from the University of Melbourne to conduct an evaluation of the Lead Site Project [19]. This paper aimed to (1) investigate whether mental health services commissioned by Australia’s 31 Primary Health Networks provided accessible care and increased reach to children and youth across Australia, and (2) identify the challenges of, and facilitating factors to, implementing services for youth with, or at risk of, severe mental illness (i.e., youth enhanced services) in 10 PHNs which acted as mental health reform leaders (i.e., Lead Sites). It describes the children (0–11 years) and youth (12–25 years) receiving mental health services commissioned by the 31 PHNs from 1 July 2016 to 31 December 2017 and outlines the services they received. Furthermore, it discusses challenges of, and facilitating factors to, implementing youth enhanced services experienced by key stakeholders from the 10 Lead Sites. This includes the three Lead Sites specifically focusing on youth enhanced services.

The authors acknowledge that some people with lived experience of mental illness prefer the terms ‘consumer’, ‘client’ or ‘service user’. However, this paper uses the term ‘consumer’ to reflect the terminology most commonly used by PHN staff and their stakeholders.

## Methods

We used data from various national minimum datasets to capture information on child and youth consumers and the services they received from all 31 PHNs. We also conducted consultations with Lead Site staff and regional stakeholders in Lead Site regions, and drew on observational data from two Lead Site meetings which specifically focused on the implementation and delivery of youth enhanced services [19]. These data sources are described in more detail below. Approval was obtained from the Human Ethics Sub-Committee at the University of Melbourne (1749426).

### Data sources

#### Routinely collected data

We used data from consumers who had one or more service sessions recorded between 1 July 2016 and 31 December 2017. These data were collected via the following purpose-designed web-based minimum datasets, into which service providers and PHNs enter de-identified data: The Primary Mental Health Care minimum data set (PMHC MDS, for services since 1 July 2017), the ATAPS minimum data set (for services prior to 1 July 2017) and the *headspace* dataset (for *headspace* services commissioned through the PHNs only). The ATAPS and *headspace* data were mapped onto the PMHC MDS [20]. We only used data from those consumers who had consented to their deidentified data being provided to the Department of Health.

Data were available at three different levels: per consumer (each row represents an individual consumer), per episode of care (each row is a series of service sessions during a continuous period of time, referred to as an ‘episode of care’) and per session (each row represents one service session event). Session-level data can be aggregated to episode-level data and episode-level data can be aggregated to consumer-level data. For the purposes of this paper, episode-level data were aggregated to individual consumers using the most recent episode of care.

#### Stakeholder consultations

Consultations were held with PHN staff from Lead Sites and regional stakeholders (e.g., individual providers or representatives of services) from their regions. The consultations were conducted via separate Lead Site staff and regional stakeholder focus groups, telephone interviews and written responses to pre-determined questions about the planning and implementation of youth enhanced services (see Appendix). All participants were provided with a plain language statement, an informed consent form and a set of demographic questions. All consultations were video- or audio-recorded.

Ten Lead Site focus groups were held between September and December 2017. Lead Site representatives received the consultation questions and were asked to invite those staff best able to answer the questions to participate. All focus groups were 2 to 3.5 h in duration. One focus group was conducted via Zoom, an online platform for video conferencing. All other focus groups were conducted face to face. All recordings were professionally transcribed verbatim.

The regional stakeholder consultations took place in March 2018. Lead Site representatives were asked to provide contact details for 10 to 20 external regional stakeholders directly working with their PHN. Regional stakeholders who were not able to attend focus groups were offered the option of participating in an individual telephone interview or providing a written response. Six focus groups were conducted via Zoom and were 1 to 2 h in duration. Two 30-min individual telephone interviews were also held. Notes were taken during verbal consultations and recordings were used to add to these notes as needed. Eight stakeholders provided a written response.

Transcripts, notes and written responses from all consultations were used for analysis.

#### Observational data

Two members of the evaluation team attended two Lead Site ‘youth enhanced’ meetings in September 2017 and March 2018. These meetings focused specifically on the implementation and delivery of youth enhanced services and were attended by the three Lead Sites specifically focusing on such services. Key points from these meetings were noted. These notes and associated documentation, including meeting minutes and two presentations, were used for analysis.

### Data analyses

#### Routinely collected data

Data from the PMHC MDS (augmented with mapped ATAPS and *headspace* data) were analysed in SPSS (Version 25), using standard descriptive statistical procedures.

#### Data from stakeholder consultations and observational data

Transcripts from Lead Site staff and regional stakeholders consultations were coded with an initial template based on pre-determined questions [21]. Using a thematic analysis approach, two members of our team independently coded one transcript, iteratively creating a coding template. Once the final set of broad themes was constructed, transcripts were re-examined, and narrower themes were identified. The two team members compared and revised the coding template until consensus was reached. During this process additional themes were identified. The final template with the complete set of broad and narrow themes was applied across all relevant stakeholder data. The three Lead Sites with a focus on youth enhanced services and other Lead Sites were compared for key differences in emerging themes.

## Results

### Receipt of PHN-commissioned services by children and youth

Table 1 gives an overview of the number of children and youth that received services and the number of episodes and sessions they received between July 2016 and December 2017.

**Table 1:** Overview of number of consumers, episodes and sessions between July 2016 and December 2017

	Children (0–11 years)		Youth (12–25 years)	
	Frequency	%	Frequency	%
Number of consumers	11,627	9.7	108,743	90.3
Number of episodes	12,160	9.5	115,879	90.5
Number of sessions	55,372	13.7	348,173	86.3

In the 18 months between July 2016 and December 2017, 11,627 children and 108,743 youth received PHN-commissioned services. Table 2 summarises the socio-demographic and clinical characteristics of the children and youth who received PHN-commissioned mental health services during the period of interest. Most children receiving services were male, while most youth were female. Up to 10% of young people receiving services were Aboriginal and/or Torres Strait Islander young people. Over half of children and youth lived in major cities and approximately 40% lived in inner or outer regional Australia. Most children and youth lived in areas of greater social disadvantage. For the majority of children and youth their diagnosis was unknown, or they either did not have a formal diagnosis or experienced subsyndromal mental health problems.

**Table 2:** Socio-demographic and clinical characteristics of children and youth receiving mental health services

	Children (0–11 years)		Youth (12–25 years)	
	Frequency	%	Frequency	%
Consumer characteristics	(n = 11,627)		(n = 108,743)	
Gender				
Female	4874	41.9	62,572	57.5
Male	6696	57.6	39,658	36.5
Other	6	0.1	1321	1.2
Unknown	51	0.4	5192	4.8
Aboriginal and/or Torres Strait Islander				
Yes	1172	10.1	9618	8.8
No	7384	63.5	87,178	80.2
Unknown	3071	26.4	11,947	11.0
Remoteness area				
Major cities of Australia	6585	56.6	65,374	60.1
Inner regional Australia	2933	25.2	28,425	26.1
Outer regional Australia	1748	15.0	12,559	11.5
Remote Australia	217	1.9	17,36	1.6
Very remote Australia	94	0.8	503	0.5
Unknown	50	0.4	146	0.1
Social disadvantage^1^				
High	3103	26.7	22,475	20.7
High to moderate	3007	25.9	23,804	21.9
Moderate	2486	21.4	24,973	23.0
Moderate to low	1823	15.7	18,472	17.0
Low	1158	10.0	18,851	17.3
Unknown	50	0.4	168	0.2
Diagnosis				
Anxiety disorders	1527	13.1	19,510	17.9
Affective disorders	197	1.7	17,661	16.2
Substance use disorders	3	0.0	762	0.7
Psychotic disorders	8	0.1	429	0.4
Disorders of childhood and adolescence	721	6.2	2343	2.2
Other mental disorders	270	2.3	3005	2.8
Subsyndromal problems/no formal diagnosis	5649	48.6	36,048	33.1
Unknown	3252	28.0	28,985	26.7

^1^Based on the index of Relative Socio-economic Disadvantage (IRSD)

A total of 12,160 episodes of care were received by children and 115,879 episodes of care were received by youth. Table 3 summarises the service characteristics over the 18-months initial period. Professional referrals mostly came from GPs and youth mostly self-referred. For most youth the episodes of care, treatment was concluded, while for children this was mostly unknown. For children most episodes compromised of 5 or less sessions (62.1%), for youth most episodes compromised of 4 or less sessions (63.3%). This was similar for the treatment-concluded episodes only.

**Table 3:** Service characteristics of children and youth receiving mental health services

	Children (0–11 years)		Youth (12–25 years)	
	Frequency	%	Frequency	%
Episode-level characteristics	(n = 12,160)		(n = 115,879)	
Referrer profession				
General practitioner	10,359	85.2	49,642	42.8
Psychiatrist	21	0.2	157	0.1
Obstetrician	0	0.0	2	0.0
Pediatrician	424	3.5	94	0.1
Other medical specialist	8	0.1	19	0.0
Midwife	2	0.0	14	0.0
Maternal health nurse	4	0.0	36	0.0
Psychologist	151	1.2	123	0.1
Mental health nurse	10	0.1	89	0.1
Social worker	38	0.3	102	0.1
Occupational therapist	0	0.0	10	0.0
Aboriginal health worker	1	0.0	19	0.0
Educational professional	93	0.8	50	0.0
Early childhood service worker	60	0.5	4	0.0
Other	62	0.5	9682	8.4
N/A-self referral	98	0.8	52,750	45.5
Unknown	829	6.8	30,86	2.7
Completion status				
Open (not completed)	2367	19.5	5387	4.6
Treatment concluded	799	6.6	65,288	56.3
Treatment closed for other reasons^1^	1075	8.8	2156	1.9
Unknown	7919	65.1	43,048	37.1
Attended sessions				
1	1809	14.9	34604	29.9
2	1550	12.7	17,075	14.7
3	1486	12.2	12,013	10.4
4	1363	11.2	9660	8.3
5	1346	11.1	7988	6.9
6 or more	4606	37.9	34,539	29.8

Session characteristics	(n = 55,372)		(n = 348,173)	
Session modality				
Face to face	53,550	96.7	331,588	95.2
Telephone	1048	1.9	15,145	4.3
Video	592	1.1	287	0.1
Internet-based	182	0.3	1153	0.3
Session participants				
Individual consumer	36,971	66.8	326,204	93.7
Consumer group	1697	3.1	14,892	4.3
Family/ support network	16,333	29.5	3877	1.1
Other health professional or service provider	88	0.2	1292	0.4
Other	17	0.0	103	0
Unknown	266	0.5	1805	0.5
Session duration				
1–15 min	475	0.9	10,626	3.1
16–30 min	1036	1.9	27,161	7.8
31–45 min	1859	3.4	2814	0.8
46–60 min	46,879	84.7	220,477	63.3
61–120 min	5049	9.0	81,880	24.0
> 120 mins	74	0.1	5132	1.5
Unknown	0	0.0	83	0.0

^1^Other reasons included: consumer couldn’t be contacted, declined further contact, moved out of the area, was referred elsewhere, or an unknown reason

**Fig. 1:**
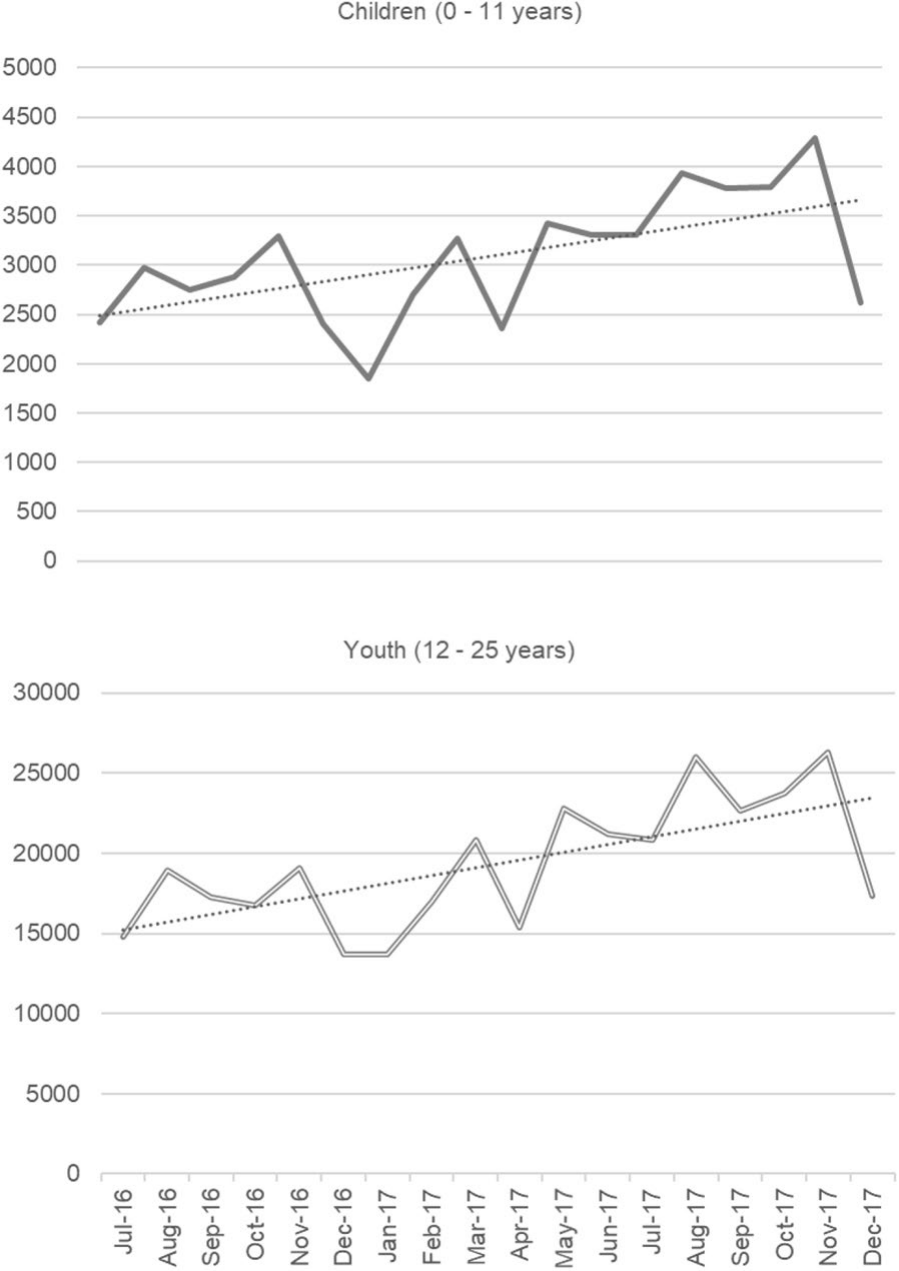
Mental health service contacts for children and youth from July 2016 to December 2017. A different scale on the Y-axis was used for each graph

In total, the children’s episodes of care comprised 55,372 sessions and the youth’s comprised 348,173. Most sessions were face to face, provided on an individual basis, and lasted between 46–60 min. Figure 1 presents the number of service sessions for children and youth from 1 July 2016 to 31 December 2017. There was an overall modest increase in service uptake of PHN-commissioned services over time, with some pronounced drops (e.g., December 2016 and April 2017).

### Stakeholder perspectives

#### Sample and demographic information

A total of 58 Lead Site staff participated in focus groups, ranging from four to 10 staff members per Lead Site. Most participants were female (81%) and aged between 30 and 49 years (62.1%), and none of the participants were Aboriginal and/or Torres Strait Islanders. The groups generally comprised a senior PHN mental health manager and a person representing each portfolio within the mental health stream (e.g., managers or program officers for youth mental health, suicide prevention, or intake). Some participants had broader responsibility for mental health services in general. Others were responsible for evaluation and research, data and planning, policy and system re-development.

In total, 62 regional stakeholders participated in consultations (four to 12 per Lead Site region), mostly of whom participated via focus groups (83.9%). Other were either interviewed by phone (3.2%) or proved written responses (12.9%). Most participants were female (53.2%) and aged between 40 and 59 years (67.7%), and none of the participants were Aboriginal and/or Torres Strait Islander. Most stakeholders were managers, chief executive officers or employees from regional service provider agencies. Some participants were consumer and carer representatives, representatives from local health departments and professional or peak bodies, independent consultants, researchers or service providers.

Staff from the Lead Sites briefly described their youth enhanced services which are summarised in Table 4. At the time of the consultations, staff from one Lead Site reported they had not yet commissioned youth enhanced services. Six key themes emerged from Lead Site staff and regional stakeholder consultations (summarised in Table 5): service access and gaps, workforce and expertise, funding and guidance, integrated and flexible service models, service promotion, and data collection, access and sharing. No clear differences emerged between the three Lead Sites with a focus on youth enhanced services and other Lead Sites.

**Table 4:** Youth enhanced services delivered by the 10 Lead Sites

Type of service or specific target group^1^
Assertive outreach services
Services for posttraumatic stress disorder
Services for homeless youth
Services for Indigenous youth
Services for youth disengaged from education
Family-focused services
Service navigation support
Improving access to psychological therapies for youth
Services for those ‘falling through the gaps’ between *headspace* and acute services

^1^Categories are not mutually exclusive

**Table 5:** Key themes from Lead Site staff and regional stakeholder consultations

Key theme	Description	Example stakeholder statement
Service access and gaps	Several stakeholders raised challenges related to a lack service access and/or existing service gaps, such as intensive (psychiatric) services and services for youth with more complex issues	'When we refer youth with more complex/severe issues, there are big waiting lists, and we get [youth with] increasing complexity. The complex cases we can only refer to hospitals'
Workforce and expertise	Stakeholders mentioned challenges with recruiting a specialised workforce and workforce retention. Some stakeholders noted it is beneficial when new services are established from organisations with existing expertise	'I think it’s good the funding went to the local health district, that’s where skills and expertise are, it’s commendable for the PHN to have chosen to do this'
Funding and guidance	Stakeholders commented on a lack of clear guidance on how to use funding, unclear key performance indicators and data requirements. They also noted that short-term funding contributed to several other challenges (e.g. workforce issues)	'There were many questions about key performance indicators, data reporting et cetera'
Integrated and flexible service models	Stakeholders pointed out the need for more integrated and flexible services for youth, including clinical and non-clinical services	'From a clinical perspective it makes sense to specialise, for youth it doesn’t’ work. It’s not what they need'
Promotion of services	Most Lead Site staff mentioned that minimal service promotion was needed. Some deliberately did not promote their services in order to manage demand	'[…] being able to share the data, the difficulty with these groups is that the data is so poor. Research team member: Does that mean planning is more difficult? Yes absolutely'
Data collection, access and sharing	Lead Site staff noted the collection of meaningful data was challenging. Better access to data and sharing of information between services was considered to be necessary for service planning and implementation	'[…] being able to share the data, the difficulty with these groups is that the data is so poor. *Research team member*: Does that mean planning is more difficult? Yes absolutely'

#### Service access and gaps

Both Lead Sites staff and regional stakeholders raised a lack service access and/or existing service gaps as a current challenge. During focus groups, staff from all 10 Lead Sites noted that intensive (psychiatric) services are needed for youth with severe and complex mental illness, but are often unavailable. Some Lead Sites and/or their service providers commented they had been able to overcome this challenge by capitalising on relationships with other services (e.g., tertiary services or private psychiatrists).

Regional stakeholders noted a variety of issues related to a lack of access to services, or service gaps, for youth. Three regional stakeholders from three Lead Sites expressed concerns about lack of access in regional areas, particularly for Lesbian Gay Bisexual Transgender and Intersex (LGTBI) and Aboriginal and/or Torres Strait Islander youth. Lack of appropriate workforce (e.g., Aboriginal and/or Torres Strait Islander youth workers) played a role in this. Several regional stakeholders from two Lead Sites commented on existing service gaps for specific groups of youth with, or at risk of, mental illness including those with intellectual impairment and a diagnosis on the Autism spectrum, those aged 18–25 years; and those with complex needs who are not eligible for acute services. Additionally, one regional stakeholder noted that the loss of other mental health programs (e.g., the Mental Health Nurse Incentive Program^1^) will create additional service gaps. Two regional stakeholders from two Lead Sites noted issues around demand being greater than supply for those with more complex issues (exemplified by the stakeholder statement in Table 5).

Several regional stakeholders mentioned challenges around hard-to-engage or disengaged youth (e.g., homeless youth and those disengaged from school or current services), with two regional stakeholders from two Lead Sites specifically mentioning the need for outreach services for disengaged youth with complex needs.

#### Workforce and expertise

Workforce challenges were mentioned by staff from nine Lead Sites during the focus groups and were reiterated by staff from the three enhanced Lead Sites and their service providers. Staff from four Lead Sites mentioned the challenges of recruiting a specialised workforce, which caused delays in program implementation. Workforce retention was also of particular concern. Workforce issues were exacerbated in PHN regions spanning large geographical areas. Three regional stakeholders from two Lead Sites indicated that outcomes for new services may be better when they are established from organisations with existing expertise and skilled staff (in addition to established referral pathways), such as Local Hospital Networks (LHNs)^2^.

#### Funding and guidance

Several regional stakeholders identified insufficient funding and guidance as a challenge. Three regional stakeholders from two Lead Sites commented on a lack of clear guidance on how to use funding. Additionally, they felt that key performance indicators and data requirements were unclear. Two regional stakeholders from one Lead Site specifically mentioned that more funding was needed. Several regional stakeholders indicated that short-term funding contributes to limited access and awareness, workforce issues, and systemic challenge of engaging GPs.

#### Integrated and flexible service models

Overall, staff from the 10 Lead Sites raised the broader challenge of ensuring services are well integrated into the broader system. Staff from two Lead Sites noted that the time pressure to develop, procure and deliver youth enhanced services was very challenging, especially for vulnerable groups.

Several regional stakeholders mentioned that youth respond better when services are co-located and integrated, operating as a ‘one stop shop’. They expressed the value of non-clinical youth programs complementing clinical services (e.g., vocational or educational support). Three regional stakeholders from two Lead Sites noted that a flexible model offering uncapped number of sessions was very helpful in fostering service engagement. Furthermore, three regional stakeholders from two Lead Sites noted that the outreach program in their region was successfully reaching youth and filling service gaps.

#### Promotion of services

Staff from several Lead Sites mentioned service promotion. Staff from three Lead Sites were using assertive outreach, and staff from one Lead Site mentioned general promotion to the public. Another Lead Site was promoting its services through non-mental health access points, such as education providers. Most Lead Site staff, however, mentioned little promotion was needed since their commissioned provider has direct access to the target group. Staff from three Lead Sites reported that they deliberately did not promote their services in order to strategically manage demand.

#### Data collection, access and sharing

Staff from several Lead Sites discussed the challenge of incentivising providers to report meaningful data. During meetings with the three youth enhanced Lead Sites, the issue of duplication of assessments was discussed. Staff from the three Lead Sites suggested this challenge could be overcome by introducing joint assessments by secondary and tertiary services, or by fostering sufficient trust between providers to rely on each other’s assessment. In this context, the importance of feedback from providers to referrers in order to build trust was noted. Access to data and sharing of information between services was thought to be needed for the planning and implementation of youth enhanced services (as exemplified by the stakeholder statement in Table 5).

## Discussion

The current findings indicate that PHN-commissioned mental health services are addressing several access barriers for children and youth. Firstly, services seemed to have improved access for certain groups of children and youth traditionally known to be underserviced. Young males typically show poor mental health service access due to perceived stigma, poor mental health literacy, masculine ideals and lack of appropriate services [22]. Current results indicate improved service uptake by boys and young males, with the majority of children receiving services being male and improved uptake (36.5%) by male youth compared to ATAPS and *headspace* in the past [7–9].

The current findings also indicate improved access for Aboriginal and Torres Strait Islander young people. Aboriginal and Torres Strait Islander young people represent approximately 5% of the Australian youth population [23]. They are more likely to experience mental health issues compared to non-Indigenous young people [23]. Current findings showed approximately 9% of children and youth who accessed services were Aboriginal and/or Torres Strait Islander young people, which shows an improvement in service uptake compared to ATAPS and *headspace* in the past [7–9].

Secondly, the findings indicate low-threshold service access. Services reached young people without a formal diagnosis or whose diagnosis was unknown, and many youth self-referred. The high self-referral rate (45.5%) for youth is notable, as earlier research suggests that youth are often reluctant to seek help [1, 10, 11]. However, for both children and youth GPs were a main entry point for mental health services.

Thirdly, services reached children and youth from areas of greater disadvantage and non-metropolitan areas. The prevalence of mental health problems is likely to be higher in non-metropolitan areas and greater socio-economic disadvantage [1]. Furthermore, young men in non-metropolitan areas have typically shown lower service access [24], which may be due to perceived stigma and lack of anonymity [22]. Approximately 40–45% of children and youth who received services were residing outside of major cities, which is comparable to figures from previous research [8, 9]. Considering approximately 28% of young people reside in non-metropolitan areas [25], current results indicated those young people received services at a higher rate compared to those residing in major cities.

Fourthly, the current findings showed a modest increase in service uptake of PHN-commissioned child and youth mental health services over time, with some pronounced drops likely associated with holiday periods. The general increase in service uptake was most pronounced for youth (12–25 years). Traditionally, this group is known to have a high prevalence of mental health problems, together with a reluctance to seek help [3, 10].

The findings also indicated areas for improvement. Minimally adequate psychological treatment of Australian adults, including youth (16–85 years), has previously been described as six or more sessions of 30 min or more [26]. For children and young adolescents (9–16 years) adequate psychological treatment has previously been described as eight or more sessions [27]. Most children and youth received sessions between 46–60 min in duration or more and received less than six sessions per episode of care. The latter may partly be due to the fact some treatments were still ongoing. However, for treatment concluded episodes only results were similar. Findings indicate that services are currently not meeting the minimally adequate treatment standard. However, it should be noted that minimally adequate treatment may compromise fewer sessions for children and youth who receive complementary medication [28].

Stakeholders noted several ongoing challenges to the implementation and delivery of new youth enhanced services for young people, including current service access issues and gaps, a lack of skilled workforce, and a lack funding and guidance. These challenges were similar for the three Lead Sites with a focus on youth enhanced services and the other Lead Sites, and were consistent with those reported for the development of child and adolescent mental health services worldwide [1].

The current results indicate that the implementation and delivery of youth mental health services in the future should involve sufficient and long-term funding which will enable improved service access and help address ongoing workforce issues (e.g. retention), build on existing expertise and skilled staff, have the ability to offer flexible and integrated service model, include meaningful data collection and facilitate data sharing between stakeholders. The ability to offer flexible and integrated service models was exemplified by outreach programs as a way of engaging hard-to-reach young people and improving service access, providing integrated clinical and non-clinical services, service co-location or a ‘one-stop-shop’, as well as providing an uncapped number of sessions depending on young people’s individual needs. Stakeholders noted that they had been able to (partly) overcome a lack of psychiatric services by capitalising on relationships with other services, highlighting the importance of local service linkages. This was further highlighted by the success of co-locating services and the need for integrated data collection and/or information sharing. This indicates that the ability to be innovative and flexible in response to region-specific mental health needs is a key to success. This is in line with former research posing that best practice for youth (mental) health includes flexible and more innovative service provision (i.e., outreach, after-hour services and various service delivery modes), and service models integrating clinical and non-clinical services [29, 30].

In order to further advance youth mental health service provision, ongoing program evaluation is needed. Firstly, future research is needed to establish the impact of various service provision approaches on both consumers and local stakeholders (e.g. outreach programs, co-located services and data sharing). Secondly, current youth mental health programs may be assessed against the best practice approaches, such as the extent of service integration that is established and the amount of flexibility in program delivery (e.g. number of sessions and modes of delivery).

### Limitations

The above findings should be interpreted in the light of several limitations. Overall, it should be noted that the Lead Sites and their regional stakeholders were still in the early stages of planning, developing, and implementing youth mental health services at the time of data collection. The current paper focused on data from the first 18 months of the introduction of the Lead Site Project to examine early implementation stages of the Lead Site Project and therefore stakeholder experiences beyond this timeframe may have changed.

Several limitations regarding the minimum datasets should also be noted. First, service providers and PHNs entered the service data into the minimum datasets and their compliance with data requirements was unknown. Second, it is likely that the number of consumers, episodes and session recorded was an underestimate, as the number of consumers who have not consented to their de-identified data being provided was unknown to the research team. However, the custodian of mental health data for all 31 PHNs reported that the data they provided to the researchers for the overall Lead Site Project evaluation represented approximately 85% of all consumers of mental health services commissioned by PHNs. Third, it is possible that there is some duplication across (and within) the minimum datasets. There were also various limitations associated with the stakeholder consultations. First, some Lead Sites experienced high staff turnover, meaning that some stakeholders may have been less aware of the Lead Site approaches and activities undertaken by the Lead Site. Second, some Lead Sites were further along the implementation and delivery stages than others, impacting on their ability to comment on the approaches and activities undertaken by the Lead Site. For example, staff from one Lead Site reported they had not yet commissioned youth enhanced services. This may be partly related to the high staff turnover experienced by some PHNs. Third, since the Lead Site representatives were asked to provide contact details for the regional stakeholders, there may have existed biases in the selection of participants. Fourth, some stakeholders may have had less capacity to participate due to inflexible work commitments (e.g., individual service providers). However, by providing the opportunity for individual interviews and written responses we tried to accommodate participation for a wide range of stakeholders. Fifth, stakeholders may have been subject to socially desirable answers during consultations. However, the mix of positive and negative views indicate genuine responses.

## Conclusion

The current paper describes children and youth who are receiving PHN-commissioned mental health services, and characteristics of the services received. Early findings suggest that these services provide accessible care and increasingly reach children and youth across Australia. The main challenges and facilitating factors for the planning and implementation of new youth enhanced services indicate that innovative and flexible service models in response to local mental health needs may be a key to success.

## Data Availability

The data that support the findings of this study are available from the Australian Government Department of Health, but restrictions apply, and the data are not publicly available. Data are available from the authors upon reasonable request and with permission of the Australian Government Department of Health.

## Appendix: Consultation questions

The following questions were used for the Lead Site staff consultations:

1. What approaches did your PHN establish to develop and commission services for young people with severe mental illnesses?
2. What specific planning activities did your PHN undertake to support development of services for this target group?
3. What types of services did your PHN develop to better meet the needs of young people experiencing severe illness?
4. How were these services procured?
5. What difficulties were faced?
6. How were the difficulties addressed?
7. How were these services targeted to ensure that they met priority service gaps identified in the regional mental health plans?
8. What planning arrangements were set up with other youth service providers in the region?
9. What selection criteria were developed to inform referrers?
10. How were these services promoted?
11. How were young people with mental health problems encouraged to use services?
12. What activities and approaches were found to be effective in delivering services to young people with severe mental illness?
13. Are there examples of where clinical care is being effectively complemented by vocational, educational and parental support programmes?
14. What effective linkages were formed with other regional youth-specific services, including those provided by states and territories, headspace, schools and other educational institutions?
15. Describe the factors that facilitated developing new services for young people with severe mental illness.
16. What were the barriers to developing new services for young people with severe mental illness?
  - Did any of these barriers significantly compromise referral arrangements, client intake and/or delivery?
  - How did your PHN deal with these barriers?

The questions for the regional stakeholders included:

1. How were you/your organisation involved in PHNs commissioning services for youth with, or at risk of, severe mental illness? For which PHN?
2. How has clinical care for young people with, or at risk of, severe mental illness been complemented by other programs important for young people, such as vocational, educational and parental support programs?
3. How might services for young people with, or at risk of, severe mental illness be improved in the future?

## Abbreviations

ATAPS: Access to Allied Psychological Services
GP: General practitioner
LGTBI: Lesbian Gay Bisexual Transgender and Intersex
LHNs: Local Hospital Networks
MDS: Minimum data set
PHNs: Primary Health Networks
PMHC MDS: Primary Mental Health Care minimum data set

## Footnotes

1 The Mental Health Nurse Incentive Program funds general practices, private psychiatric services and other appropriate community providers to employ mental health nurses to help provide coordinated clinical care for people in the community with severe mental illness.

2 LHNs are entities established by state and territory governments to manage single or small groups of public hospitals, including managing budgets and being directly responsible for performance.
